# Ivarmacitinib reduces the need for adding/escalating medications in moderate-to-severe rheumatoid arthritis patients: a *post hoc* analysis from a phase III trial

**DOI:** 10.3389/fphar.2025.1683508

**Published:** 2025-11-21

**Authors:** Huaxiang Liu, Jie Li, Lijun Song

**Affiliations:** Department of Rheumatology, Qilu Hospital, Shandong University, Jinan, Shandong, China

**Keywords:** ivarmacitinib, rheumatoid arthritis, high selective JAK inhibitor, adding/escalating medication, phase III trial

## Abstract

**Background:**

Uncontrolled rheumatoid arthritis (RA) requires increasing the dosage of medications or adding combination therapies, leading to higher costs and increased risks of adverse events. This study aimed to assess the impact of ivarmacitinib, a selective Janus kinase 1 inhibitor, on the needs for adding/escalating medications in patients with moderate-to-severe RA.

**Methods:**

This was a *post hoc* study from a phase III clinical trial (NCT04333771). Patients were randomized to receive ivarmacitinib 4 mg (n = 189), 8 mg (n = 189), or placebo (n = 188) until week 24 (W24). From W24 to W52, patients with placebo switched to ivarmacitinib 4 mg, while patients with ivarmacitinib continued the initial treatment. Adding/escalating medication was defined as an increased dosage or new medication addition for RA treatments (excluding the study drug).

**Results:**

Ivarmacitinib 4 mg (7.4%) and 8 mg (5.3%) groups had significantly lower rates of adding/escalating medication compared to the placebo group (22.3%) within W24 (both *P* < 0.001). Specifically, the ivarmacitinib groups presented lower rates of adding/escalating oral glucocorticoids (1.1% and 0.5%, versus 5.9%) and non-steroidal anti-inflammatory drugs (6.9% and 4.2%, versus 20.2%) than the control group within W24. No statistical significance was observed between groups in adding/escalating intravenous/intramuscular corticosteroids, conventional synthetic disease-modifying antirheumatic drugs, or systemic immunosuppressants. From W24 to W52, the rates of adding/escalating medications remained low in ivarmacitinib 4 mg (4.2%) and 8 mg (3.2%) groups; the switched group showed a reduced rate of adding/escalating medications (12.2%).

**Conclusion:**

Ivarmacitinib significantly reduces the need for adding/escalating medications compared to placebo, thereby potentially decreasing treatment burden. However, the *post hoc*, exploratory nature of this study requires further validation for the findings.

## 1 Introduction

Rheumatoid arthritis (RA) is an autoimmune disease with typical symptoms of joint swelling, stiffness, and pain (Di Matteo et al., 2023). The ultimate goal of treatment for RA is to achieve a low disease activity or remission, thus reducing the risk of disability and improving patient’s quality of life (Fraenkel et al., 2021; Singh, 2022). For patients with uncontrolled disease under treatment, adding/escalating medications through combining drugs or elevating the dosage of medication is often needed (Smolen et al., 2023; Smolen et al., 2020; Tian et al., 2024). However, considering that patients with RA usually require long-term treatment, adding/escalating medications would increase the risk of adverse events and financial burden for patients (Hsieh et al., 2020; Sepriano et al., 2023; So et al., 2023; Vinson et al., 2020).

Ivarmacitinib (SHR0302) is a highly selective Janus kinase (JAK) 1 inhibitor, which has demonstrated efficacy in immune-mediated inflammatory diseases including ankylosing spondylitis, atopic dermatitis, alopecia areata, and ulcerative colitis (Chen et al., 2022; Zhao et al., 2025; Zhou et al., 2023). A phase III clinical trial further reported that in patients with moderate-to-severe RA, both ivarmacitinib 4 mg and 8 mg significantly improved the American College of Rheumatology response criteria (ACR20, ACR50, and ACR70) compared with placebo at week 24. Additionally, both doses of ivarmacitinib reduced disease activity and promoted multiple dimensions of patient-reported outcomes (PROs) compared with placebo; these benefits of ivarmacitinib were sustained through week 52 (Liu et al., 2025). However, whether JAK inhibitors such as ivarmacitinib could reduce the adding or escalating other medications for the treatment of RA remains unclear.

The phase III clinical has demonstrated the efficacy of ivarmacitinib in patients with moderate-to-severe RA during the core period (24 weeks) and in the long term (52 weeks). This *post hoc* analysis retrieved the 52-week data from the phase III randomized clinical trial to investigate the impact of ivarmacitinib treatment on adding/escalating medications in patients with moderate-to-severe RA within 52 weeks.

## 2 Materials and methods

### 2.1 Study design

This was a *post hoc* analysis of a phase III multicenter, randomized, double-blind clinical trial (NCT04333771) (Liu et al., 2025), which evaluated ivarmacitinib in patients with moderate-to-severe active RA who had inadequate response to conventional synthetic disease-modifying antirheumatic drugs (csDMARDs). Specifically, this analysis assessed the impact of ivarmacitinib on the requirement for adding/escalating RA medications. The study protocol was approved by the Ethics Committee of all participating institutions, and written informed consent was obtained from all participants.

### 2.2 Patients

A total of 566 patients were randomized to one of three treatment arms: 4 mg ivarmacitinib (n = 189), 8 mg ivarmacitinib (n = 189), or placebo (n = 188). During the initial 24-week period, patients maintained their assigned regimens. At W24, placebo recipients were switched to 4 mg ivarmacitinib, while patients receiving active treatment continued their original regimens throughout week 52 (W52).

### 2.3 Adding/escalating medications

Adding/escalating medication was defined as either dose escalation of existing drugs or adding new therapeutic agents. Monitored medication categories included: csDMARDs, oral glucocorticoids, intravenous/intramuscular corticosteroids, systemic immunosuppressants, and non-steroidal anti-inflammatory drugs (NSAIDs). Both the incidence (from W0 to W24 and from W24 to W52) and cumulative incidence (from W0 to W52) of adding/escalating medication were evaluated.

In the phase III clinical trial, investigators were allowed to adjust the concomitant treatment for RA if a patient showed less than 20% improvements in tender and swollen joint counts after 12 weeks of treatment. The primary choice was adjustments to NSAIDs, acetaminophen, or weak opioids. csDMARDs, oral glucocorticoids, intravenous/intramuscular corticosteroids, and others could be considered if the response was still inadequate. More details about adding/escalating medications were mentioned in the previous study (Liu et al., 2025).

### 2.4 Statistics

Statistical analyses were performed using SPSS 29.0 (IBM, United States) from November 2024 to February 2025. Continuous variables are presented as mean ± standard deviation (SD), while categorical variables are expressed as counts (percentages). Differences in rates with 95% confidence interval (CI) were also presented. Group comparisons employed t-tests for continuous variables and χ^2^ tests for categorical variables, with no adjustment for multiple comparisons. Missing data were treated with non-responder imputation (NRI). Kaplan-Meier curve with log-rank test were applied for accumulating incidences of adding/escalating medications for treatment of RA. A two-tailed *P* value <0.05 was considered statistically significant.

## 3 Results

### 3.1 Baseline characteristics

The baseline characteristics of the three groups are reported previously (Liu et al., 2025). The placebo, ivarmacitinib 4 mg, and ivarmacitinib 8 mg groups had a mean age of around 50 years, with over 80% of patients being females. The RA duration of the three groups was over 9 years. In the placebo, ivarmacitinib 4 mg, and ivarmacitinib 8 mg groups, 97.9%, 95.2%, and 97.4% patients were using csDMARDs at baseline; while the rates were 44.7%, 37.0%, and 45.5% for NSAIDs. The rates of baseline glucocorticoids usage rates were 37.6%, 40.2%, and 30.7% in the three groups, respectively. The baseline demographic characteristics and disease features were generally balanced among the three groups (Table 1). However, the proportion of patients with hydroxychloroquine usage at baseline was lower in ivarmacitinib 4 mg group than in the placebo group.

**TABLE 1:** Demographics and baseline disease characteristics.

Items	Placebo (N = 188)	Ivarmacitinib 4 mg (N = 189)	Ivarmacitinib 8 mg (N = 189)
Age (years), mean ± SD	50.9 ± 9.8	49.7 ± 10.4	49.8 ± 10.9
Sex, No. (%)			
Male	27 (14.4)	17 (9.0)	31 (16.4)
Female	161 (85.6)	172 (91.0)	158 (83.6)^c^
BMI (kg/m^2^), mean ± SD	22.8 ± 3.4	22.5 ± 3.4	22.9 ± 3.3
Smoke status, No. (%)			
Never	171 (91.0)	176 (93.1)	166 (87.8)
Former	5 (2.7)	3 (1.6)	5 (2.6)
Current	12 (6.4)	10 (5.3)	18 (9.5)
Drink status, No. (%)			
Never	180 (95.7)	180 (95.2)	170 (89.9)
Former	3 (1.6)	4 (2.1)	9 (4.8)
Current	5 (2.7)	5 (2.6)	10 (5.3)
Time since RA diagnosis (years), mean ± SD	9.9 ± 7.3	10.1 ± 8.0	9.2 ± 7.4
ACR functional class, No. (%)			
I	21 (11.2)	27 (14.3)	23 (12.2)
II	118 (62.8)	105 (55.6)	116 (61.4)
III	49 (26.1)	57 (30.2)	50 (26.5)
csDMARDs use at baseline, No. (%)	184 (97.9)	180 (95.2)	184 (97.4)
Methotrexate	107 (56.9)	104 (55.0)	100 (52.9)
Leflunomide	72 (38.3)	74 (39.2)	79 (41.8)
Hydroxychloroquine	39 (20.7)	24 (12.7)^a^	25 (13.2)
Iguratimod	14 (7.4)	16 (8.5)	16 (8.5)
Sulfasalazine	6 (3.2)	7 (3.7)	5 (2.6)
Chloroquine	0 (0.0)	1 (0.5)	0 (0.0)
NSAIDs, No. (%)	84 (44.7)	70 (37.0)	86 (45.5)
Oral glucocorticoid use, No. (%)	69 (36.7)	76 (40.2)	58 (30.7)
Previous bDMARDs received, No. (%)	50 (26.6)	52 (27.5)	43 (22.8)
CRP (mg/L), mean ± SD	15.5 ± 17.7	18.7 ± 20.0	19.4 ± 18.9^b^
ESR (mm/hour), mean ± SD	44.5 ± 24.1	48.2 ± 27.7	47.5 ± 23.6
RF-positive, No. (%)	158 (84.0)	155 (82.0)	155 (82.0)
Anti-CCP-positive, No. (%)	169 (89.9)	166 (87.8)	170 (89.9)
SJC, mean ± SD	12.1 ± 6.4	12.9 ± 7.8	11.9 ± 6.0
TJC, mean ± SD	20.9 ± 11.6	21.1 ± 14.1	20.7 ± 13.1
PtGA (0–100 mm VAS), mean ± SD	61.3 ± 19.0	60.3 ± 21.0	61.3 ± 18.9
PGA (0–100 mm VAS), mean ± SD	61.6 ± 14.3	60.9 ± 15.7	61.6 ± 15.3
Pain (0–100 mm VAS), mean ± SD	59.6 ± 18.7	57.5 ± 21.0	58.1 ± 19.5
DAS28(CRP), mean ± SD	5.2 ± 0.8	5.2 ± 0.9	5.3 ± 0.9
DAS28 (ESR), mean ± SD	6.2 ± 0.9	6.2 ± 1.0	6.3 ± 0.9
CDAI, mean ± SD	35.4 ± 10.5	35.0 ± 12.2	35.1 ± 11.7
SDAI score, mean ± SD	37.0 ± 10.8	36.8 ± 12.8	37.1 ± 12.4
HAQ-DI, mean ± SD	1.2 ± 0.7	1.2 ± 0.6	1.1 ± 0.6
SF-36 PCS, mean ± SD	42.1 ± 10.8	40.8 ± 10.8	41.8 ± 10.9
SF-36 MCS, mean ± SD	35.5 ± 6.7	35.8 ± 7.4	35.6 ± 7.0
Morning stiffness duration (min), mean ± SD	52.7 ± 63.7	64.1 ± 81.9	61.6 ± 82.2
Morning stiffness severity (0–100 mm VAS), mean ± SD	45.7 ± 24.1	45.4 ± 25.7	46.5 ± 25.5

^a^ *P* < 0.05 for ivarmacitinib 4 mg vs. placebo.

^b^ *P* < 0.05 fr ivarmacitinib 8 mg vs. placebo.

^c^ *P* < 0.05 fr ivarmacitinib 4 mg vs. ivarmacitinib 8 mg.

RA, rheumatoid arthritis; SD, standard deviation; BMI, body mass index; ACR, american college of rheumatology; csDMARDs, conventional synthetic disease-modifying antirheumatic drugs; NSAIDs, non-steroidal anti-inflammatory drugs; bDMARDs, biologic disease-modifying antirheumatic drugs; CRP, C reactive protein; ESR, erythrocyte sedimentation rate; RF, rheumatoid factor; anti-CCP, anticyclic citrullinated protein antibody; SJC, swollen joint count; TJS, tender joint count; PtGA, patient global assessment of disease activity; PGA, physician global assessment of disease activity; DAS28, Disease Activity Score 28; CDAI, clinical disease activity index; SDAI, simplified disease activity index; HAQ-DI, health assessment questionnaire disability index; SF-36 PCS, Short Form-36 physical component summary; SF-36 MCS, Short Form-36 mental component summary.

### 3.2 Comparison of total adding/escalating medications among groups

**FIGURE 1:**
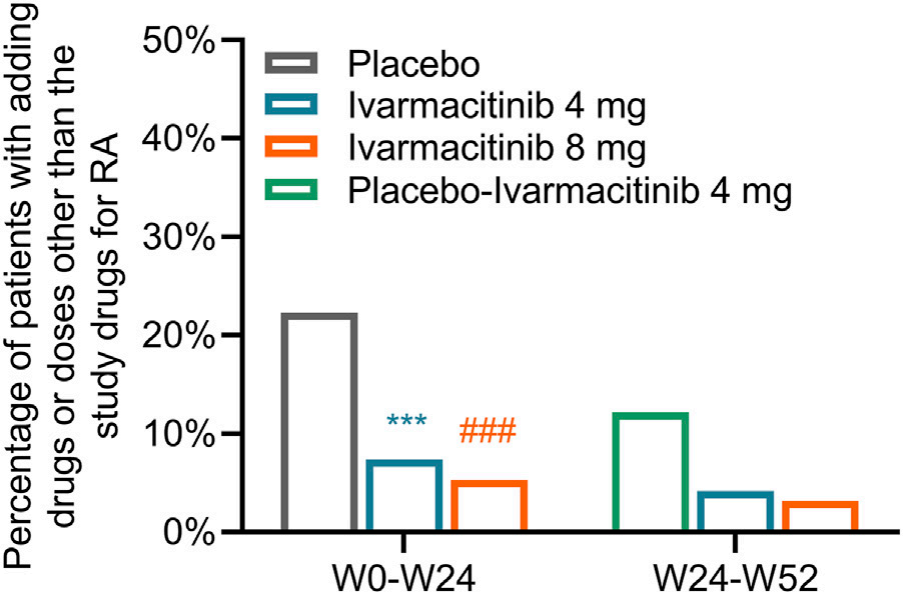
Total incidence of adding/escalating medications within W24 and from W24 to W52. ***: *P* < 0.001 in ivarmacitinib 4 mg group compared with the placebo group; ###: *P* < 0.001 in ivarmacitinib 8 mg group compared with the placebo group.

From W0 to W24, 42 (22.3%) patients in the placebo group experienced adding/escalating medications. Meanwhile, 14 (7.4%) and 10 (5.3%) patients in ivarmacitinib 4 and 8 mg groups underwent adding/escalating medications, which were significantly lower than in the placebo group (both *P* < 0.001). The differences in the rates of adding/escalating medications were −14.9% (95% CI: −22.1% ∼ −7.6%) and −17.0% (95% CI: −24.0% ∼ −10.1%) in ivarmacitinib 4 and 8 mg groups compared with the placebo group. From W24 to W52, the incidence of adding/escalating medications remained low in ivarmacitinib 4 mg (4.2%) and ivarmacitinib 8 mg (3.2%) groups. Moreover, adding/escalating medications were 12.2% in the placebo-ivarmacitinib 4 mg group (Figure 1).

### 3.3 Comparison of detailed categories of adding/escalating medications among groups

From W0 to W24, adding/escalating oral glucocorticoids and NSAIDs were significantly higher in the placebo compared to both ivarmacitinib 4 mg and 8 mg groups. In detail, the incidence of adding/escalating oral glucocorticoids was 1.1% in ivarmacitinib 4 mg group and 0.5% in ivarmacitinib 8 mg group compared with 5.9% in the placebo group (both *P* < 0.05). The difference in rates of adding/escalating oral glucocorticoids were −0.05% (95% CI: −0.08% ∼ −0.01%) and −0.05% (95% CI: −0.09% ∼ −0.02%) in ivarmacitinib 4 mg and 8 mg groups compared with the placebo group. The incidence of adding/escalating NSAIDs was 6.9% in ivarmacitinib 4 mg group and 4.2% in ivarmacitinib 8 mg group versus 20.2% in the placebo group (both *P* < 0.001). The difference in rates of adding/escalating NSAIDs were −13.3% (95% CI: −20.2% ∼ −6.4%) and −16.0% (95% CI: −22.6 ∼ −9.4%) in ivarmacitinib 4 and 8 mg groups compared with the placebo group. However, the incidence of adding/escalating csDMARDs, intravenous/intramuscular corticosteroids, and systemic immunosuppressants was low (≤0.5% in each group); no difference was found between ivarmacitinib groups and the placebo group (all *P* > 0.05). From W24 to W52, both ivarmacitinib 4 and 8 mg groups remained low incidences of adding/escalating csDMARDs, oral glucocorticoids, intravenous/intramuscular corticosteroids, systemic immunosuppressants, and NSAIDs. In placebo-ivarmacitinib 4 mg group, the incidences of adding/escalating csDMARDs, intravenous/intramuscular corticosteroids, and systemic immunosuppressants remained low; notably, the incidence of adding/escalating of NSAIDs was 4.3% (Table 2).

**TABLE 2:** Patients with adding drugs or doses other than the study drugs for RA.

Items	W0-W24			W24-W52		
	Placebo	Ivarmacitinib 4 mg	Ivarmacitinib 8 mg	Placebo-ivarmacitinib 4 mg	Ivarmacitinib 4 mg	Ivarmacitinib 8 mg
All, No. (%)	42 (22.3)	14 (7.4)^b^	10 (5.3)^d^	23 (12.2)	8 (4.2)	6 (3.2)
csDMARDs, No. (%)	1 (0.5)	0 (0.0)	1 (0.5)	1 (0.5)	0 (0.0)	1 (0.5)
Oral glucocorticoids, No. (%)	11 (5.9)	2 (1.1)^a^	1 (0.5)^c^	13 (6.9)	3 (1.6)	1 (0.5)
Intravenous/intramuscular corticosteroids, No. (%)	1 (0.5)	0 (0.0)	0 (0.0)	1 (0.5)	2 (1.1)	0 (0.0)
Systemic immunosuppressants, No. (%)	0 (0.0)	0 (0.0)	0 (0.0)	1 (0.5)	0 (0.0)	0 (0.0)
NSAIDs, No. (%)	38 (20.2)	13 (6.9)^b^	8 (4.2)^d^	8 (4.3)	4 (2.1)	4 (2.1)

^a^ *P* < 0.05 for ivarmacitinib 4 mg vs. placebo.

^b^ *P* < 0.001 for ivarmacitinib 4 mg vs. placebo.

^c^ *P* < 0.01 for ivarmacitinib 8 mg vs. placebo.

^d^ *P* < 0.001 for ivarmacitinib 8 mg vs. placebo.

RA, rheumatoid arthritis; W12, 12 weeks; W24, 24 weeks; csDMARDs, conventional synthetic disease-modifying antirheumatic drugs; NSAIDs, non-steroidal anti-inflammatory drugs.

**FIGURE 2:**
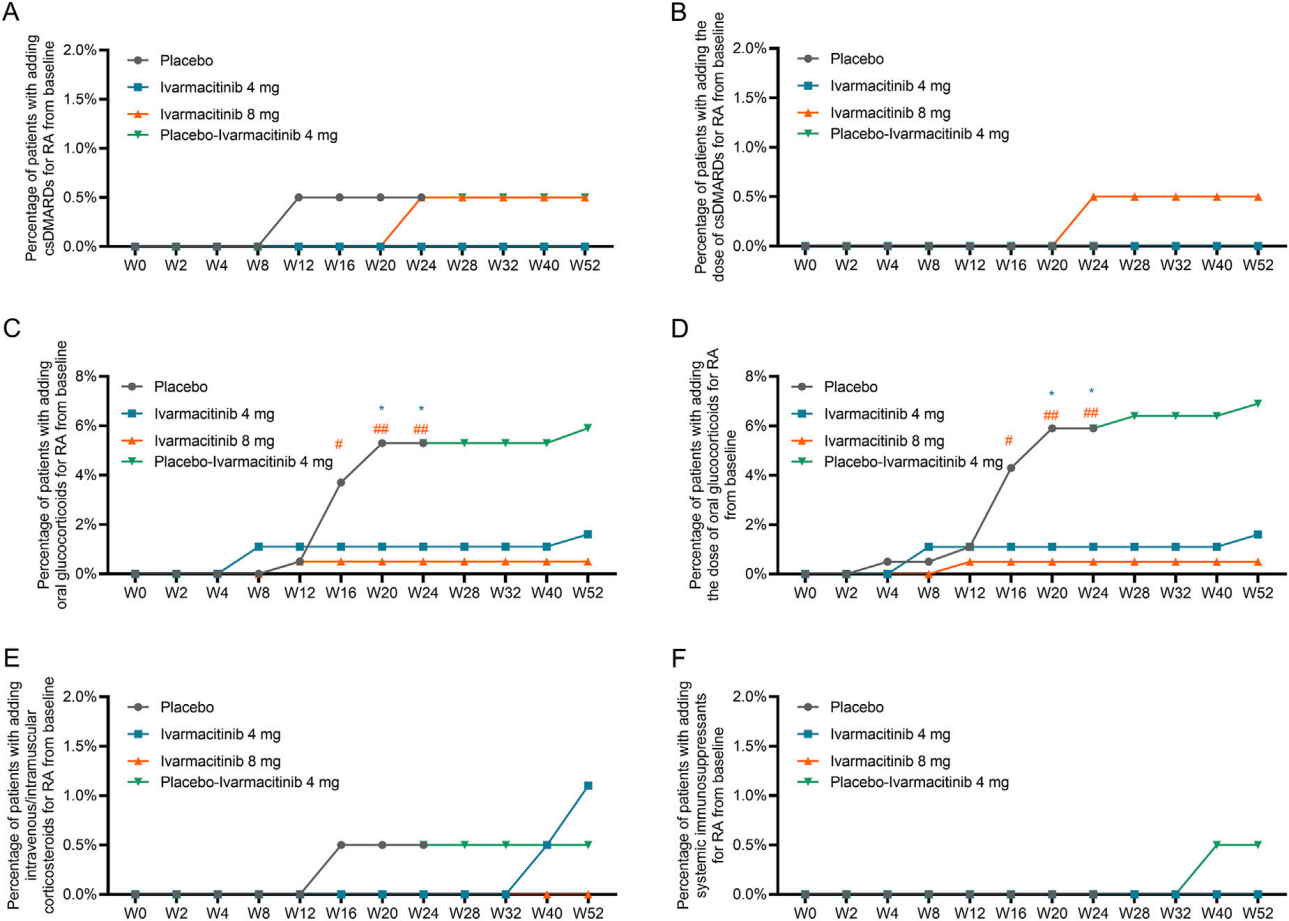
Accumulating incidence of adding/escalating csDMARDs, oral glucocorticoids, and intravenous/intramuscular corticosteroids from W0 to W52. Accumulating incidence of adding csDMARDs **(A)** and escalating csDMARDs **(B)**. Accumulating incidence of adding oral glucocorticoids **(C)** and escalating oral glucocorticoids **(D)**. Accumulating incidence of adding intravenous/intramuscular corticosteroids **(E)** and escalating intravenous/intramuscular corticosteroids **(F)**. *: *P* < 0.05 in ivarmacitinib 4 mg group compared with the placebo group; #: *P* < 0.05, ##: *P* < 0.01 in ivarmacitinib 8 mg group compared with the placebo group.

The cumulative incidence (from W0) of adding or escalating oral glucocorticoids was significantly lower in ivarmacitinib 8 mg group at W16, W20, and W24, as well as in ivarmacitinib 4 mg group at W20 and W24 versus placebo group (all *P* < 0.05). However, no significant differences were observed among groups in cumulative incidence of adding or escalating csDMARDs or intravenous/intramuscular corticosteroids. At each timepoint between W24 and W52, the cumulative incidence (from W0) of adding or escalating oral glucocorticoids, csDMARDs, and intravenous/intramuscular corticosteroids were generally well-maintained in the three groups (Figures 2A–F). The detailed cumulative incidences of adding/escalating medications in the three groups are shown in Supplementary Table S1.

**FIGURE 3:**
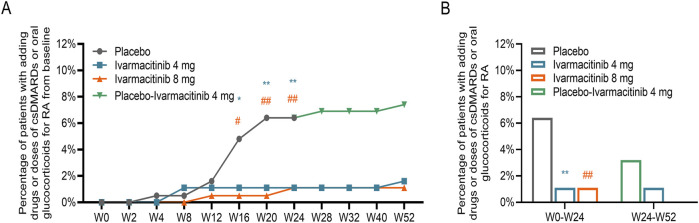
Adding/escalating csDMARDs or oral glucocorticoids. Accumulating incidence of adding/escalating csDMARDs or oral glucocorticoids from W0 to W52 **(A)**. Total incidence of adding/escalating csDMARDs or oral glucocorticoids within W24 and from W24 to W52 **(B)**. *: *P* < 0.05, **: *P* < 0.01 in ivarmacitinib 4 mg group compared with the placebo group; #: *P* < 0.05, ##: *P* < 0.01 in ivarmacitinib 8 mg group compared with the placebo group.

From W0 to W52, cumulative incidence of adding/escalating csDMARDs or oral glucocorticoids is shown in Figure 3A. Within W24, significantly lower accumulating incidences (from W0) of adding/escalating csDMARDs or oral glucocorticoids were noticed in ivarmacitinib 4 and 8 mg groups at W16, W20, and W24 compared with the placebo group (all *P* < 0.05). During W24 to W52, cumulative incidence of adding/escalating csDMARDs or oral glucocorticoids was well-maintained in the three groups. Moreover, the incidence of adding/escalating csDMARDs or oral glucocorticoids was lower in ivarmacitinib 4 and 8 mg groups than in the placebo group within W24 (both *P* < 0.01) (Figure 3B).

The Kaplan-Meier curves showed that only the accumulating incidence of adding/escalating glucocorticoids was lower in ivarmacitinib 4 mg (*P* = 0.011) and 8 mg (*P* = 0.003) groups compared with the placebo group. While the accumulating incidences of adding/escalating other medications were not different among groups (Supplementary Figure S1A–E).

Generally, ivarmacitinib reduced adding/escalating medications in patients with moderate-to-severe RA within 24 weeks, mainly glucocorticoids and NSAIDs. From week 24–52, all groups showed low incidences of adding/escalating medications.

## 4 Discussion

Adding/escalating medications during treatment are associated with higher risk of adverse events and increased financial burden in patients with RA (He et al., 2022; Ntais et al., 2024; Sen et al., 2024; Sepriano et al., 2023). Using the data from the phase III clinical trial, this study compared the condition of adding/escalating medications between RA patients receiving ivarmacitinib and those with placebo. The data revealed that within W24, both ivarmacitinib 4 mg and 8 mg significantly reduced the needs for adding/escalating medications compared with placebo, respectively. A possible explanation was that a higher needs for adding/escalating medications in the placebo group suggested worse disease control in these patients; while ivarmacitinib achieved better control of disease activity compared with placebo. This was also revealed by the phase III clinical trial, in which the primary endpoint of ACR20 response reached 70.4% in ivarmacitinib 4 mg group and 75.1% in the 8 mg group versus 40.4% in the placebo group (Liu et al., 2025). Therefore, fewer patients in the ivarmacitinib 4 and 8 mg groups needed adding/escalating medications than those in the placebo group. The lower incidence of adding/escalating medications by ivarmacitinib could lead to several potential benefits, such as lower risk of adverse events and financial burden. However, further studies were needed for verification.

Different category of drugs for RA treatment would induce different adverse events. csDMARDs, such as methotrexate, are associated with gastrointestinal symptoms, liver enzyme abnormality, and mucocutaneous adverse events (Sherbini et al., 2021). Long-term glucocorticoid administration can induce cardiovascular events, infections, and decreased bone mineral density (Blavnsfeldt et al., 2018; Doumen et al., 2023). Similarly, NSAIDs use is also known to induce gastrointestinal adverse events (Wang et al., 2011). In the current study, the analysis revealed that ivarmacitinib 4 and 8 mg significantly decreased the need of adding/escalating oral glucocorticoids and NSAIDs compared with placebo within W24. However, adding/escalating csDMARDs, intravenous/intramuscular corticosteroids, or systemic immunosuppressants remained low and were not different among ivarmacitinib 4 mg, ivarmacitinib 8 mg, and placebo groups. A possible explanation was that oral glucocorticoids and NSAIDs are primarily considered under the condition of uncontrolled disease activity (Tian et al., 2024). Meanwhile, NSAIDs and glucocorticoids were the most commonly prescribed drugs in patients with RA according to a previous study (Gaujoux-Viala et al., 2023). Therefore, ivarmacitinib might reduce the risks of adverse events associated with the use of glucocorticoids and NSAIDs in patients with RA (Bergstra et al., 2023; Roubille et al., 2015). Until present, no study investigated the effect of JAK inhibitors on adding/escalating medications in patients with moderate-to-severe RA. Therefore, further studies should be conducted to verify the findings of this study.

In the phase III clinical trial of ivarmacitinib in RA, patients with placebo were given ivarmacitinib 4 mg from W24 to W52, and switching from placebo to ivarmacitinib improved treatment response, disease activity, and patient-reported outcomes (Liu et al., 2025). This study further demonstrated that during W24 to W52, the need for adding/escalating medications decreased substantially in the placebo-ivarmacitinib 4 mg group. These findings highlighted the benefit of switching from placebo to ivarmacitinib. Moreover, the incidence of adding/escalating medications remained low in ivarmacitinib 4 and 8 mg groups during W24 to W52. These findings indicated that ivarmacitinib possessed the ability of continuously reducing the needs of adding/escalating medications.

Moreover, the current *post hoc* study highlighted the potential of ivarmacitinib to reduce the needs of adding/escalating medications, which could serve as evidence supporting the application of ivarmacitinib in patients with moderate-to-severe RA. However, several limitations should be clarified. First, all patients included were Chinese, which hindered the generalizability of the findings. Second, the incidence of adding/escalating NSAIDs was not collected in detail at each assessment timepoint, which partly weakened the findings regarding the effect of ivarmacitinib on adding/escalating NSAIDs. Third, the treatment duration was limited to 52 weeks, and the effect of ivarmacitinib on long-term adding/escalating medications should be further investigated. Fourth, the current study only investigated the needs of adding/escalating medications after receiving ivarmacitinib or placebo. However, dose reductions for background medications in continuous use were not documented. Fifth, the *post hoc* nature of this study could induce potential bias. Sixth, the long-term safety profile of ivarmacitinib should be investigated in further studies. Seventh, no multiple comparison was performed, which raise the concern of type I error.

## 5 Conclusion

In conclusion, ivarmacitinib consistently reduces the needs of adding/escalating medications particularly oral glucocorticoids and NSAIDs in patients with moderate-to-severe RA. Switching from placebo to ivarmacitinib also provides notable effects on reducing the needs of adding/escalating medications in these patients. The findings of this study highlighted that ivarmacitinib could reduce the burden related to adding/escalating medications in these patients. However, this is a *post hoc*, exploratory study. Future studies with larger sample size, diverse populations, real-world data, and long-term follow-up are needed to verify our findings.

## Data Availability

The original contributions presented in the study are included in the article/Supplementary Material, further inquiries can be directed to the corresponding author.
