# Supplementary material for: Ivarmacitinib reduces the need for adding/escalating medications in moderate-to-severe rheumatoid arthritis patients: a post hoc analysis from a phase III trial
Source: Front Pharmacol. 2025 Nov 21;16:1683508. doi: 10.3389/fphar.2025.1683508 (PMC12678364; doi:10.3389/fphar.2025.1683508)
Supplement: Supplementary file 1 [file Supplementaryfile1.docx]

**Supplementary Table 1.** Cumulative percentage of patients with adding/escalating medications

|  | Group | W0 (%) | W2 (%) | W4 (%) | W8 (%) | W12 (%) | W16 (%) | W20 (%) | W24 (%) | W28 (%) | W32 (%) | W40 (%) | W52 (%) |
| --- | --- | --- | --- | --- | --- | --- | --- | --- | --- | --- | --- | --- | --- |
| Adding csDMARDs | Placebo/Placebo-Ivarmacitinib 4 mg | 0.0 | 0.0 | 0.0 | 0.0 | 0.5 | 0.5 | 0.5 | 0.5 | 0.5 | 0.5 | 0.5 | 0.5 |
|  | Ivarmacitinib 4 mg | 0.0 | 0.0 | 0.0 | 0.0 | 0.0 | 0.0 | 0.0 | 0.0 | 0.0 | 0.0 | 0.0 | 0.0 |
|  | Ivarmacitinib 8 mg | 0.0 | 0.0 | 0.0 | 0.0 | 0.0 | 0.0 | 0.0 | 0.5 | 0.5 | 0.5 | 0.5 | 0.5 |
| Escalating csDMARDs | Placebo/Placebo-Ivarmacitinib 4 mg | 0.0 | 0.0 | 0.0 | 0.0 | 0.0 | 0.0 | 0.0 | 0.0 | 0.0 | 0.0 | 0.0 | 0.0 |
|  | Ivarmacitinib 4 mg | 0.0 | 0.0 | 0.0 | 0.0 | 0.0 | 0.0 | 0.0 | 0.0 | 0.0 | 0.0 | 0.0 | 0.0 |
|  | Ivarmacitinib 8 mg | 0.0 | 0.0 | 0.0 | 0.0 | 0.0 | 0.0 | 0.0 | 0.5 | 0.5 | 0.5 | 0.5 | 0.5 |
| Adding oral glucocorticoids | Placebo/Placebo-Ivarmacitinib 4 mg | 0.0 | 0.0 | 0.0 | 0.0 | 0.5 | 3.7 | 5.3 | 5.3 | 5.3 | 5.3 | 5.3 | 5.9 |
|  | Ivarmacitinib 4 mg | 0.0 | 0.0 | 0.0 | 1.1 | 1.1 | 1.1 | 1.1 | 1.1 | 1.1 | 1.1 | 1.1 | 1.6 |
|  | Ivarmacitinib 8 mg | 0.0 | 0.0 | 0.0 | 0.0 | 0.5 | 0.5 | 0.5 | 0.5 | 0.5 | 0.5 | 0.5 | 0.5 |
| Escalating oral glucocorticoids | Placebo/Placebo-Ivarmacitinib 4 mg | 0.0 | 0.0 | 0.5 | 0.5 | 1.1 | 4.3 | 5.9 | 5.9 | 6.4 | 6.4 | 6.4 | 6.9 |
|  | Ivarmacitinib 4 mg | 0.0 | 0.0 | 0.0 | 1.1 | 1.1 | 1.1 | 1.1 | 1.1 | 1.1 | 1.1 | 1.1 | 1.6 |
|  | Ivarmacitinib 8 mg | 0.0 | 0.0 | 0.0 | 0.0 | 0.5 | 0.5 | 0.5 | 0.5 | 0.5 | 0.5 | 0.5 | 0.5 |
| Adding intravenous/intramuscular corticosteroids | Placebo/Placebo-Ivarmacitinib 4 mg | 0.0 | 0.0 | 0.0 | 0.0 | 0.0 | 0.5 | 0.5 | 0.5 | 0.5 | 0.5 | 0.5 | 0.5 |
|  | Ivarmacitinib 4 mg | 0.0 | 0.0 | 0.0 | 0.0 | 0.0 | 0.0 | 0.0 | 0.0 | 0.0 | 0.0 | 0.0 | 1.1 |
|  | Ivarmacitinib 8 mg | 0.0 | 0.0 | 0.0 | 0.0 | 0.0 | 0.0 | 0.0 | 0.0 | 0.0 | 0.0 | 0.0 | 0.0 |
| Adding systemic immunosuppressants | Placebo/Placebo-Ivarmacitinib 4 mg | 0.0 | 0.0 | 0.0 | 0.0 | 0.0 | 0.0 | 0.0 | 0.0 | 0.0 | 0.0 | 0.5 | 0.5 |
|  | Ivarmacitinib 4 mg | 0.0 | 0.0 | 0.0 | 0.0 | 0.0 | 0.0 | 0.0 | 0.0 | 0.0 | 0.0 | 0.0 | 0.0 |
|  | Ivarmacitinib 8 mg | 0.0 | 0.0 | 0.0 | 0.0 | 0.0 | 0.0 | 0.0 | 0.0 | 0.0 | 0.0 | 0.0 | 0.0 |

csDMARDs, conventional synthesized disease-modifying antirheumatic drugs
