# Supplementary figures and images for: Ivarmacitinib reduces the need for adding/escalating medications in moderate-to-severe rheumatoid arthritis patients: a post hoc analysis from a phase III trial
Source: Front Pharmacol. 2025 Nov 21;16:1683508. doi: 10.3389/fphar.2025.1683508 (PMC12678364; doi:10.3389/fphar.2025.1683508)

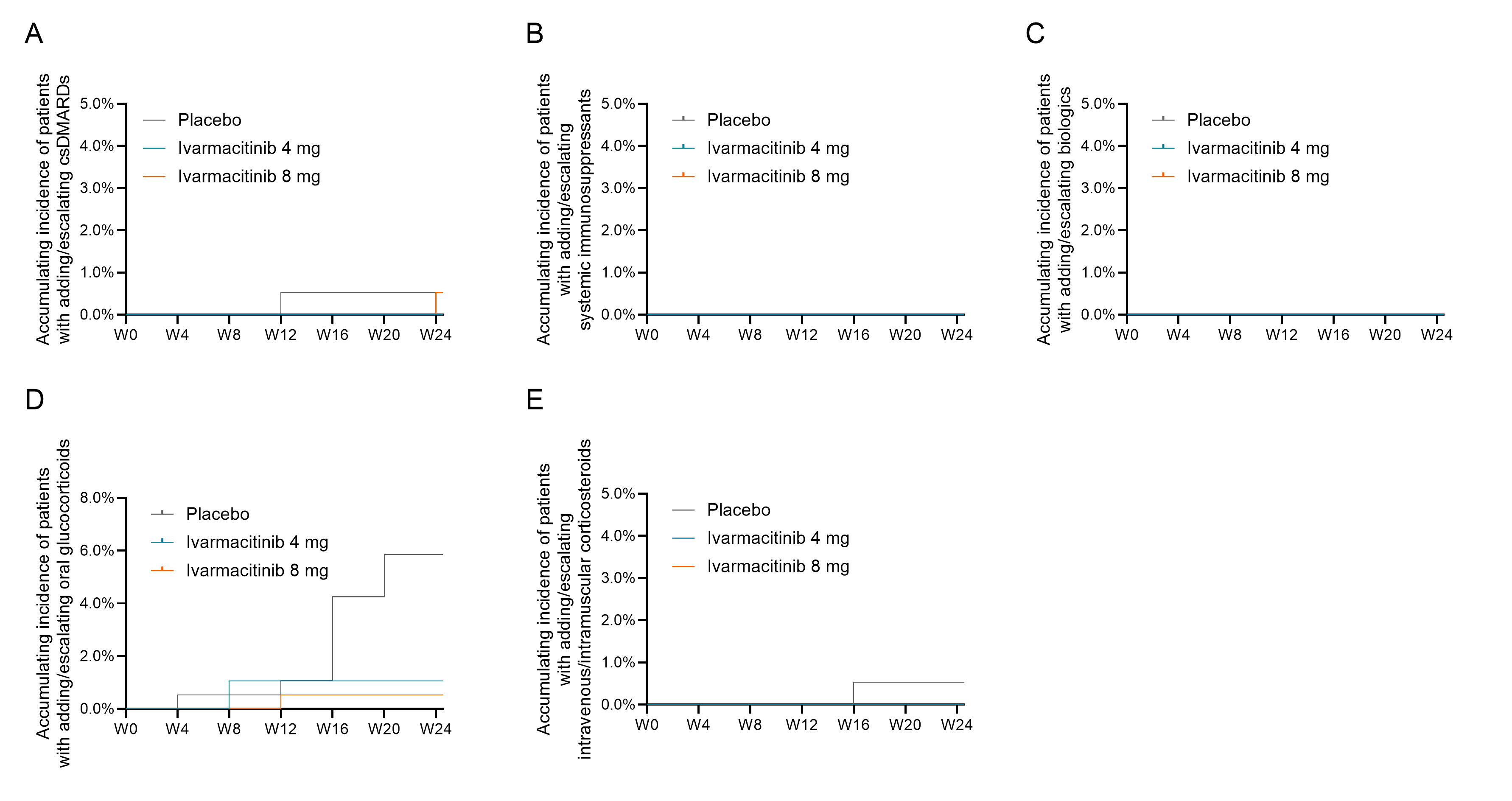

Supplement: Supplementary file 2 [file Image1.tif]
